# Assessment of codivergence of Mastreviruses with their plant hosts

**DOI:** 10.1186/1471-2148-8-335

**Published:** 2008-12-18

**Authors:** Beilei Wu, Ulrich Melcher, Xingyi Guo, Xifeng Wang, Longjiang Fan, Guanghe Zhou

**Affiliations:** 1 State Key Laboratory for Biology of Plant Diseases and Insect Pests, Institute of Plant Protection, Chinese Academy of Agricultural Sciences, No. 2 West Yuanming Rd, Beijing 100193, PR China; 2 Department of Biochemistry and Molecular Biology, Oklahoma State University, Stillwater, OK 74078-3035, USA; 3 College of Agriculture and Biotechnology, Zhejiang University, Hangzhou 310029, PR China

## Abstract

**Background:**

Viruses that have spent most of their evolutionary time associated with a single host lineage should have sequences that reflect codivergence of virus and host. Several examples for RNA viruses of host-virus tree congruence are being challenged. DNA viruses, such as mastreviruses, are more likely than RNA viruses to have maintained a record of host lineage association.

**Results:**

The full genomes of 28 isolates of *Wheat dwarf virus *(WDV), a member of the *Mastrevirus *genus, from different regions of China were sequenced. The analysis of these 28 entire genomes and 18 entire genome sequences of cereal mastreviruses from other countries support the designation of wheat, barley and oat mastrevirus isolates as separate species. They revealed that relative divergence times for the viruses WDV, Barley dwarf virus (BDV), Oat dwarf virus (ODV) and Maize streak virus (MSV) are proportional to divergence times of their hosts, suggesting codivergence. Considerable diversity among Chinese isolates was found and was concentrated in hot spots in the Rep A, SIR, LIR, and intron regions in WDV genomes. Two probable recombination events were detected in Chinese WDV isolates. Analysis including further *Mastrevirus *genomes concentrated on coding regions to avoid difficulties due to recombination and hyperdiversity. The analysis demonstrated congruence of trees in two branches of the genus, but not in the third. Assuming codivergence, an evolutionary rate of 10^-8 ^substitutions per site per year was calculated. The low rate implies stronger constraints against change than are obtained by other methods of estimating the rate.

**Conclusion:**

We report tests of the hypothesis that mastreviruses have codiverged with their monocotyledonous hosts over 50 million years of evolution. The tests support the hypothesis for WDV, BDV and ODV, but not for MSV and other African streak viruses.

## Background

Viruses are a class of genetic elements dependent on suitable host cells for their propagation. Viruses belonging to diverse viral groups have been proposed to have codiverged with their hosts based on congruence of phylogenetic trees for the viruses with those for their hosts. In codivergence, congruence results from long association of the viral and host lineages. The term codivergence is preferred to describe this situation since, unlike the term "coevolution", it does not imply that the association necessarily provides mutual benefits to the partners [1].

The best studied examples of codivergence [2] include the hantaviruses [3] and arenaviruses [4,5] in their murid hosts, and potyviruses [6] and tobamoviruses [7,8] in plant hosts. Three of these examples have recently been challenged. A reanalysis of hantaviruses, including data on shrew hantaviruses, has called the codivergence of hantaviruses with hosts into question [9]. The role of recombination in generating arenavirus phylogenetic trees is under dispute [10]. A recent reanalysis of *Potyvirus *divergence suggested that this genus emerged shortly after the beginning of agriculture [11], much later than was earlier proposed. The recent analysis of the evolution of rymoviruses [12] supports the view that evolution of sobemoviruses is more rapid than that of their hosts. In contrast, the addition of further tobamoviral sequences to the *Tobamovirus *tree has supported the congruence of host and virus trees [13]. Further, there is evidence that, in very long-term analyses, such as through studies of viruses in herbarium specimens [14] and Greenland ice cores [15], viruses in the genus are evolving extremely slowly, such that codivergence is a possibility, while having nucleotide substitution frequencies of the order of 10^-5 ^substitutions per site in the shorter term [16].

The viruses in these major examples of putative codivergence of viruses and hosts have RNA genomes that replicate using error prone RNA-dependent RNA polymerases encoded by the viral genomes. Such viruses are expected to evolve more rapidly than viruses with DNA genomes which use host DNA-dependent DNA polymerases with proof-reading ability for replication of their genomes and could be subject to the action of DNA repair systems on replication errors or spontaneous mutations [17]. Thus, for DNA-containing viruses, mutation frequencies similar to those of host genomes are expected, making the observation of codivergence more likely for these viruses than for viruses with RNA genomes.

Members of the *Geminiviridae *replicate their DNA using a host DNA polymerase and encapsidate circular single-stranded DNAs [17]. This plant virus family is one of the largest, represented by four genera: *Mastrevirus*, *Curtovirus*, *Topocuvirus *and *Begomovirus*, classified depending on their vectors, host range and genomic characteristics [18-20]. During the last two decades these viruses have emerged as devastating pathogens, threatening crop production and causing huge economic losses [20]. Today, geminivirus-induced diseases are among the most economically important in vegetable and field crops, including beans, cassava, cotton, maize, pepper, tomato and wheat [20-24].

In the process of studying populations from China of *Wheat dwarf virus *(WDV) from the *Mastrevirus *genus of *Geminiviridae*, we observed patterns that suggested that viruses in this genus have substitution frequencies consistent with their replication by host DNA polymerases. The genus *Mastrevirus *consists of viruses with circular single-stranded (ss) DNA genomes in geminate (twinned) virions [19], and has 11 recognized species including WDV. WDV is transmitted in a persistent circulative manner by the leafhopper *Psammotetix striatus *L. to barley, wheat, oats, rye and many wild grasses [25,26]. It was first described by Vacke [27] in the western parts of the former Czechoslovak Socialist Republic (CSR) and then found in many parts of the world [27,28]. Its distribution areas are increasing and it has recently been detected in Germany [29], Tunisia [30], Turkey [28], Finland [31], Zambia [28] and China [32]. The complete genome sequences of 18 isolates, 10, 7 and 1 from wheat, barley and oats respectively, have been determined from the CSR, Sweden, Hungary, France, Germany, Turkey and China [25]. Comparisons of these sequences showed that the isolates which infected wheat, barley and oats respectively, formed three distinct clades [25,28]. Schubert et al. [25] suggested reclassifying WDV into three species according to sequence differences and host range studies: WDV, *Barley dwarf virus *(BDV), and *Oat dwarf virus *(ODV), designations used in this paper.

## Results

### Phylogenetic analysis of viruses

**Figure 1:**
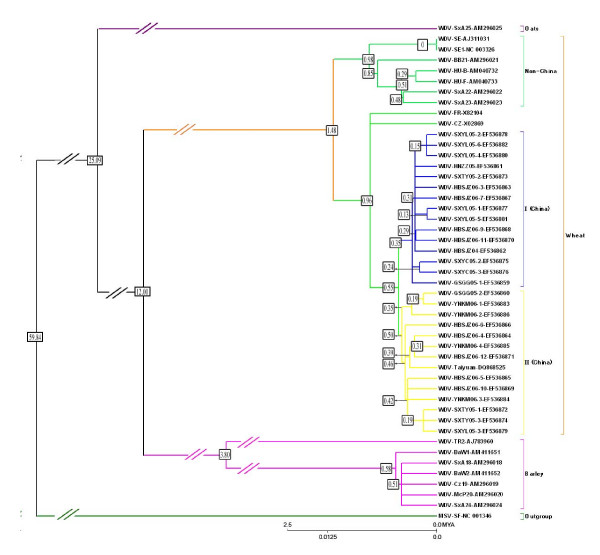
**Phylogenetic tree deduced from the complete genomic sequences of 46 WDV isolates from wheat, barley and oats produced by Neighbor Joining using the Kimura 2-parameter model**. Terminal branch colors identify two clades of isolates collected from wheat in China (yellow, blue), or in other countries (light green) and oats or barley (purple). Maize streak virus (NC_001346, dark green) was used as an outgroup.

In the spring of 2004, 2005 and 2006, several diseased wheat plants showing extreme dwarfing, various types of yellowing, and reduced or no heading were found during field surveys in many wheat fields of China [32]. Wheat samples collected from northern, central, northwestern and southwestern areas tested positive by PCR for WDV, suggesting that WDV was widely distributed throughout China. The full genomes of 28 isolates from different regions of China were sequenced in this study. Details of these, together with those of the 18 complete WDV, BDV and ODV genomes already published, are provided in supplemental material [see Additional file 1 and 2]. Phylogenetic trees were constructed by neighbor-joining (NJ) (Figure 1) and maximum-parsimony as described in Methods using *Maize streak virus *as an outgroup. The topologies of the two types of trees were identical at all branch points that were well supported by bootstrap analysis (> 70%) but differed at some branch points with low statistical support (Figure 1).

Bootstrap analysis supported an unambiguous host-dependent clustering of wheat-, barley-, and oat-derived samples. The isolates also fell into subpopulations seemingly structured according to geographic origin. Wheat isolates from China were > 94.8% identical in nucleotide sequence, whereas > 92% identities were seen between the wheat isolates of China and those of other countries. Greater than 94% nucleotide sequence identities were found among barley isolates. Nucleotide sequence identities were < 69% between wheat and barley isolates. The oat-derived isolate exhibited 59.7%–69.3% identities to those of barley, and 68.3%–69.4% to those of wheat. The values for wheat-barley, barley-oat and oat-wheat comparisons were below the threshold for *Mastreviruses *species (75%) as proposed by the ICTV [18,25].

Unscaled evolutionary distances from the common ancestors to their sequence progeny were deduced from the NJ tree. The distances indicated that the barley isolates diverged from wheat isolates much more recently than the oat isolate did. The most basal barley isolate was TR2 from Turkey, followed by those from Germany and the CSR. Wheat isolates were separated into two groups, the first one from Europe and the second one from France, the CSR, and China. The grouping of all of the Chinese isolates in one clade with the CSR and French isolates suggested that they evolved from the same ancestor. The Chinese wheat isolates were divided into two clades, but were not clustered by geographical source or collection time (Figure 1). For example, 10 isolates from Shijiazhuang, Hebei province, were classified into four different sub-clades in the phylogenetic trees (Figure 1).

### Dating divergences

Barley and wheat are members of the tribe *Triticeae*. Together with the *Aveneae*, which includes oats, they belong to the BEP clade of the *Poaceae*, while maize represents the PACCAD clade of the *Poaceae *[33]. Maize and wheat lineages have been suggested to split between 50 and 80 Mya [34] and between 44 and 60 Mya [35]. The estimate for the wheat-barley divergence from a common ancestor is 11.4 +/- 0.6 Mya [36]. The divergence of *Aveneae *(oat) and *Triticeae *(barley and wheat) has been placed at 25 Mya [37]. In the absence of provision of an error for this estimate, we assumed a range of possible times from 22 to 28 Mya. Thus, the relationships of the maize, oat, barley and wheat mastreviruses revealed in the phylogenetic tree of Figure 1 appeared to mirror the taxonomic positions of their hosts, which observation caused us to test whether their relative divergence times agreed with estimates for divergences of the host species. Relative divergence times for the respective mastreviruses were calculated by MEGA and the correspondence between host and virus times was examined by linear regression. Table 1 showed that the r^2 ^values were greater than 0.99 regardless of whether middle, high, or low values for host divergence times were used, with the middle values giving the best fit. Thus, we cannot reject the hypothesis that the mastreviruses codiverged with their hosts. Relative divergence times for viral evolution were converted to Mya times using the slope of the relationship of the middle host divergence times with virus distance, using the assumption that variation occurs according to a linear molecular clock. The results (Figure 1) suggest that the CSR, French and Chinese WDV isolates diverged from others 1.5 Mya and the Chinese isolates split off from the other two 0.4 My later. The radiation of sequences of Chinese isolates was predicted by this analysis to have occurred 0.6 Mya. Of the sites in the mastrevirus genome, 32% were substituted during evolution from the common ancestor of WDV and BDV, leading to a calculated evolutionary rate of 1 × 10^-8 ^(substitutions/site)/year.

**Table 1:** Correlation of host lineage and WDV divergence estimates.

Estimate	Maize-Wheat^a^	Oat-Triticeae^b^	Wheat-Barley^c^	r^2d^
High				
Plant	80	28	13	0.9955
Mastrevirus^e^	78	33	16	
Medium				
Plant	60	25	11.4	0.9999
Mastrevirus	60	25	12.0	
Low				
Plant	50	22	10	0.9997
Mastrevirus	50	21	10.1	

^a^Maize-wheat divergence time range of 50 to 80 Mya, with 60 Mya the most likely was from Wolfe et al. [34].

^b^Divergence of *Aveneae *from *Triticeae *assumed to occur close to the time of divergence of *Poeae *from *Triticeae *which was estimated at 25 Mya by Gaut [37].

^c^*Triticum-Hordeum *divergence estimated at 11.4 +/- 0.6 Mya by Huang et al. [36].

^d^Value from linear regression of MEGA calculated viral divergence times with host plant divergence times assuming origin at zero.

^e^Values shown are calculated from linear regression correlation and MEGA values.

### Mastrevirus sequence alignment

**Figure 2:**
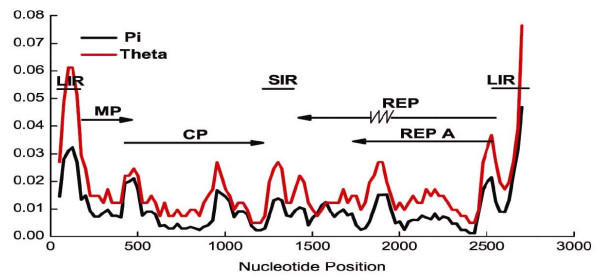
**Distribution of genetic diversity across the WDV genome based on 29 Chinese WDV isolates**. Values of Watterson's theta and of the Pi (π) estimate of the average pairwise differences between sequences in a sample were determined for windows of 100 residues evaluated every 25 residues. Diagrammed are positions of the large (LIR) and small (SIR) intergenic regions and the MP, CP, Rep and Rep A coding regions. The zig-zag line in Rep identifies the intronic region of the gene.

To explore further the possibility of the codivergence of virus and host, we included additional members of the *Mastrevirus *family in the phylogenetic analysis. To align these additional sequences with the previously analyzed sequences in an unambiguous and reliable fashion, we considered elimination of regions of high diversity and regions prone to be different due to recombination. Regions with high nucleotide diversity were difficult to align reliably. To identify regions that could be reliably aligned, the level of genetic diversity (θw and π) [38,39] of WDV was examined in genomic regions (Table 2) or along the entire genomes of Chinese isolates (Figure 2). Examination of nucleotide diversity values (Table 2) revealed few differences among the coding regions. For all WDV isolates, θw was significantly higher for the Rep region than for the other three regions, but this difference was not significant for the π nucleotide diversity. The higher Rep diversity was most apparent among non-Chinese isolates, for which both measures showed significance. Overall, as expected from Figure 1, diversity values were lower for Chinese isolates than for non-Chinese isolates. For the Chinese isolates, the π diversity values were slightly higher for Rep and RepA regions than for CP and MP regions. Theta values were not significantly different from one another. As is generally expected for mastreviruses, the most diverse regions over the 37 genomes were LIR and SIR [25,28]. Considering only the Chinese isolates, however, the diversity of the SIR region was not distinguishable from those of the coding regions. In the non-Chinese isolates, MP diversity could not be distinguished from diversity for the non-coding regions. Among the non-Chinese isolates, the SIR region was more diverse than LIR, while the opposite was true for the Chinese isolates. Overall, SIR was more diverse by the θw measure, but not significantly so using the π diversity value.

A plot of the diversity values as a function of position in the genome revealed that determinations of diversity over large genomic regions obscure regions of high diversity embedded in a background of low diversity (Figure 2). The diversity distribution presented a fluctuating pattern: (1) high diversity regions were seen in both non-coding regions and coding regions; (2) divergence in the movement protein (MP) region was low except for the extreme 5' and 3' terminal regions of it; (3) highly diverse portions were also located at the 5' terminal regions of coat protein (CP), replication-associated protein (Rep) and Rep A genes; (4) in contrast, the lower diversity portions were located in the central and 3' terminal regions of CP genes and in the overlapping part of Rep and Rep A genes; (5) there were two additional high diversity stretches in the complete genome, one was in the intron, the other was in the LIR (Table 2). The smallest nucleotide diversity along the entire genomes of WDV isolates (Table 2) was located in the CP gene, but the gene also had 5' and internal regions of elevated diversity.

**Table 2:** WDV nucleotide diversities.

Gene	Population	Theta-W	Pi(π)
CP	Total	0.01384 (0.00206)	0.01040 (0.00158)
	China	0.00752 (0.00157)	0.00414 (0.00055)
	Non-China	0.00788 (0.00197)	0.00885 (0.00109)
MP	Total	0.01656 (0.00602)	0.00881 (0.00113)
	China	0.00752 (0.00157)	0.00414 (0.00055)
	Non-China	0.01213 (0.00623)	0.00956 (0.00164)
Rep	Total	0.03817 (0.00386)	0.01433 (0.00540)
	China	0.00919 (0.00149)	0.00541 (0.00047)
	Non-China	0.05080 (0.00564)	0.03542 (0.01862)
Rep A	Total	0.01694 (0.00226)	0.00862 (0.00109)
	China	0.00932 (0.00173)	0.00504 (0.00054)
	Non-China	0.01213 (0.00243)	0.01020 (0.00148)
LIR	Total	0.10402 (0.00786)	0.05718 (0.01662)
	China	0.03673 (0.00478)	0.02082 (0.00299)
	Non-China	0.13111 (0.01120)	0.14667 (0.03912)
SIR	Total	0.20873 (0.01710)	0.10994 (0.04736)
	China	0.01184 (0.00419)	0.00573 (0.00092)
	Non-China	0.33606 (0.02753)	0.39933 (0.10137)

Numbers in parentheses are standard deviation of estimates. The 37 complete wheat WDV genomes were used.

That regions with relatively low nucleotide diversities represented protein coding regions (Figure 2) suggested that these regions evolved under negative selection. To test this hypothesis, frequencies of synonymous and non-synonymous substitutions at sites in each of the four protein coding regions were calculated. Nucleotide substitutions at non-synonymous positions of non-overlapping regions (MP and CP) were less frequent than those of overlapping regions (Rep and RepA, Table 3). The K_a_/K_s _ratios of all coding regions were lower than 0.22, which suggested negative or purifying selection (K_a_/K_s _ratios < 1 indicating purifying selection) acting on the sequences, with no genes under positive selection (K_a_/K_s _ratio > 1 indicating positive selection). The CP gene had the lowest ratio, whereas that of the Rep A gene was highest (Table 3).

**Table 3:** Nucleotide substitution for coding regions of the WDV genome.

Coding region	K_s_	K_a_	K_a_/K_s_
CP	0.03477	0.00332	0.09548
MP	0.02389	0.00394	0.16492
Rep	0.04523	0.00711	0.15718
Rep A	0.02197	0.00465	0.21165

Numbers represent the result of analysis of 37 wheat WDV genomes. A K_a_/K_s _ratio < 1 indicates purifying selection, K_a_/K_s _= 1 suggests neutral evolution, and K_a_/K_s _> 1 indicates positive selection.

**Figure 3:**
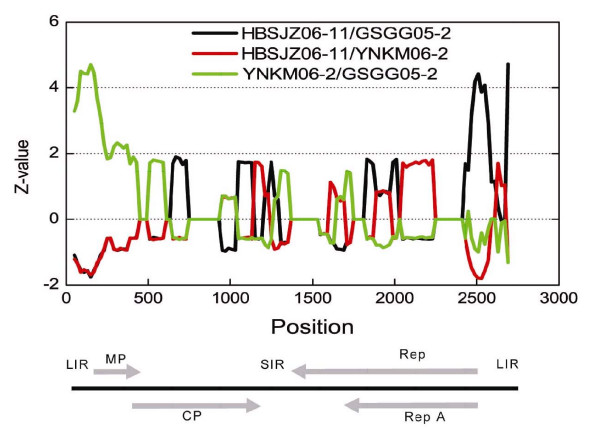
**Evidence of recombination in the evolution of Chinese WDV isolates**. SiScan analysis, as implemented in RDP3 program (v 1.5), was performed on 29 WDV genomes from China. Shown are the results of comparison of the only two isolates (HBSJZ06-11 and YNKM06-2) that yielded significant recombination signatures when compared to a reference genome, in this case GSGG05-2. The genome organization of WDV (linearized at nucleotide position 1) is shown for orientation, including the long intergenic region (LIR), the short intergenic region (SIR), and coding regions for replication-associated proteins Rep A and Rep, movement protein (MP), and coat protein (CP).

Mastreviruses are known to have experienced recombination in their evolution [40]. Phylogenetic analysis of sequences containing recombination junctions can result in misleading interpretations. Visual inspection of the alignment of Chinese WDV nucleotide sequences suggested that parts of the genomes of HBSJZ06-11 and YNKM06-2 isolates derived from a genome or genomes not included in the analysis. Analysis by algorithms contained in RDP3 [41] confirmed the visual observation. The result of SiScan [42] analysis (Figure 3) illustrates the possibility that these isolates were recombinants. The sequence of isolate GSGG05-2 was used as reference and is typical of all other Chinese WDV sequences. A Z-value (significance score) > 3 indicates reliably that two of the three sequences are more closely related to one another than either is to the third, identifying the third as a probable recombinant. HBSJZ06-11 was identified as having a recombinant segment in the LIR on one side of the nick site, while YNKM06-2 appeared to have an LIR segment derived from an unknown genome on the other side of the nick site. Since the recombined segment was nevertheless similar to WDV sequences, the recombination events likely were intraspecific. Work of others identifies the LIR and the SIR as frequent sites of recombination. Because of diversity hot spots in intergenic regions and the tendency for those regions to derive from recombination, further phylogenetic analysis was restricted to the coding regions.

### Phylogenetic comparison of viruses and hosts

**Figure 4:**
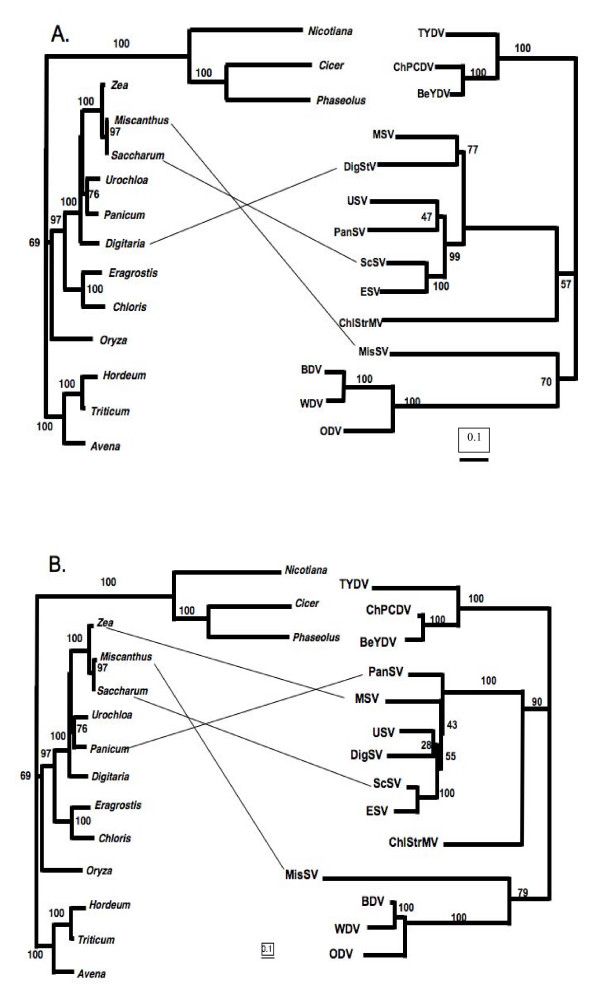
**Tangle-gram of phylogenetic relationships of host plants (left, *rbcL *gene sequences) and (right) mastreviruses infecting those plants**. A. Virus tree based on Rep and Rep A coding regions. B. Virus tree based on MP-CP coding regions. Accession numbers of the *rbcL *gene sequences used are as follows: AJ746257*Avena fatua*; EU196765*Phaseolus vulgaris*; AM235066*Miscanthus capensis*; AY632368*Panicum virgatum*; EU492898*Triticum aestivum*; AM849336*Digitaria ciliaris*; Z00044*Nicotiana tabacum*; X86563*Zea mays*; EF115541*Hordeum vulgare*; AM849409*Chloris gayana*; AP006714*Saccharum officinarum*; AM849338*Eragrostis minor*; AF308707*Cicer arietinum*; AY522330*Oryza sativa*; AM849390*Urochloa maxima*.

To facilitate alignment, consensus MP-CP and Rep-RepA sequences were generated for mastreviruses with multiple sequenced representatives (WDV, BDV, MSV, *Panicum streak virus *(PanSV), *Sugarcane streak virus *(ScSV), and *Urochloa streak virus *(USV)). Single available sequences of *Chloris striate virus (*ChlStrV), *Digitaria streak virus *(DigSV), *Miscanthus streak virus *(MisSV), *Eragrostis streak virus *(ESV), *Tobacco yellow dwarf virus *(TYDV), *Bean yellow dwarf virus *(BeYDV) and *Chickpea chlorotic dwarf virus *(ChPCDV) also were included in the alignment [see Additional file 2]. NJ distance trees were constructed for Rep-RepA (Figure 4A) and MP-CP (Figure 4B) regions. A similar distance tree was constructed from an alignment of NCBI GenBank *rbcL *gene sequences from the plastids of respective host plants for which the virus was named, recognizing that the plant named in the virus name is not necessarily the one from which the virus is most commonly isolated. The *rbcL *tree is shown in each Figure 4A and 4B to the left of the virus trees. In both Rep-RepA and MP-CP trees, the isolates named for hosts in the BEP clade formed a monophyletic cluster, consistent with branching patterns of Figure 1. Similarly, the three mastreviruses of dicotyledonous plants formed a monophyletic grouping. The branching structure within these groups mirrored precisely the structure of the *rbcL *trees of the host plants. Viruses named for hosts in the PACCAD clade were not monophyletic since MisSV was basal to the clade of BEP clade viruses in both Rep-RepA and MP-CP trees. Among the remaining PACCAD clade viruses, ChlStrV was in basal position and ESV and ScSV branched together in both viral trees. In the MP-CP tree, insufficient bootstrap support among the other PACCAD clade viruses prevented further comparison. Relationships between viruses with PACCAD host names and the hosts are not straightforward as noted by the off-vertical lines in Figure 4. A comparison of branch lengths among the Rep-RepA, MP-CP and *rbcL *trees suggests that a uniformly ticking molecular clock should not be supported. The dicotyledonous plants were on a relatively longer branch than the viruses bearing their names. MisSV had diversified more than the BEP clade viruses in the MP-CP region, but the opposite was true in the Rep-RepA region. ChlStrMV also showed anomalous branch lengths. Such anomalies are signs of recombination events after which adjustment of the recombined segments to one another is needed [8]. A series of molecular clock tests supported these observations. A clock could be rejected if all viral sequences were considered, regardless of which sequence was chosen as outgroup for the tree to be tested. However, when ChlStrV or the viruses of dicotyledonous plants were omitted with MisSV as outgroup, a clock could no longer be rejected. Operation of a clock was predicted with confidence (p > 0.9) for a dataset containing WDV, BDV, ODV, MisSV and ChlStrV with ChlStrV as outgroup.

### TreeMap analysis

**Figure 5:**
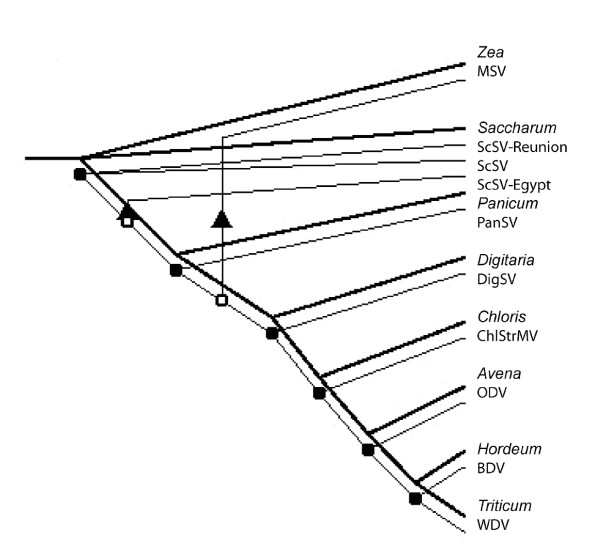
**TreeMap reconstruction of evolutionary pathways of selected *Mastrevirus *species**. Darker lines indicate the host evolutionary pathway while thinner lines indicate the pathways of the viruses. Arrowheads indicate host species jumps.

Distance trees constructed from an alignment of a subset of *rbcL *gene sequences and of the respective viruses were analyzed (Figure 5) by TreeMap [43,44]. The algorithm resulted in reconciliation of the two trees by the addition of two host species jumps by viruses (Figure 5). One was of the lineage that gave rise to the Egypt species of ScSV from a non-sugarcane lineage to the sugarcane lineage. The second jump was of MSV to maize, consistent with the long term existence of the virus in Africa before the arrival of maize in the continent [45]. The confidence level assigned to the reconciled tree was p < 0.01. Neither of the two trees had been rooted, though it is known that the host tree has its root between *Chloris *and *Avena *branches.

## Discussion

The ICTV proposed a reduced similarity value for the demarcation of species in the case of mastreviruses (75%), compared to that for the majority of geminiviruses (89%) [18]. In our analysis, we found 68% nucleotide sequence identity between wheat and barley isolates, and 59.7%–69.3% between oat and barley isolates. The nucleotide sequence identities between oat and wheat isolates were 68.3%–69.4%, similar to the results of others [25]. The phylogenetic trees also supported strongly the proposal that WDV should be divided into three mastrevirus species [25], a proposal with which we agree.

Several observations favor the hypothesis that mastreviruses codiverged with their hosts. First, despite the known propensity for mastreviruses to recombine during evolution [40], exemplified by the evidence of recombination in the evolution of two WDV isolates from China (Figure 3), the similarity of Rep-RepA and MP-CP *Mastrevirus *trees to one another (Figure 4) suggests that recombination had been eliminated as a major factor shaping trees by concentrating on the coding regions. Second, the topologies of maize, oat, barley and wheat lineages and the lineages of their mastreviruses (MSV, ODV, BDV, and WDV) were identical (Figure 1) as were the topologies of dicotyledonous plants and their viruses (Figure 4). Third, not only did the topologies agree, but there was an excellent correlation between estimated divergence times of the plant lineages and the relative divergence times of the mastreviruses, as calculated from the sequences (Table 1).

Nevertheless, support for the hypothesis of codivergence of host and virus is not conclusive. That the molecular clock has not been ticking uniformly in all lineages and in both halves of the viral genome removes the molecular clock as a tool for testing the codivergence hypothesis. Evidence for uneven ticking of the clock, such as in MisSV and ChStrMV branches, may be attributable to recombination. Yet, it is interesting that there was statistical support for a clock operating in one part of the overall tree. That part specifically included two of the branch points for which there are dates associated with divergences of plant lineages (of *Triticum *from *Hordeum *and of *Triticeae *from *Aveneae*). It is also noteworthy that adding the third datable branch (PACCAD clade from BEP clade) led to a near perfect correlation of host divergence times with relative viral divergence times (Table 1).

Non-congruence of PACCAD clade host branching patterns with branching patterns of viruses named for those hosts seems to argue against the codivergence hypothesis. However, it must be remembered in this regard that virus names are based on the host plant from which the virus was first isolated. Thus, the names do not necessarily reflect the plant lineage in which the virus spent most of its time evolving. Indeed, non-congruence is expected for viruses that can efficiently infect many species of plants. MSV isolates have been found in many grass genera including *Zea, Panicum, Setaria, Urochloa *and even *Triticum *[40]. Further, their presence in maize is of recent origin since the streak viruses are indigenous to Africa and Indian Ocean islands and maize was only introduced to Africa after the European discovery of America [45]. Similarly, sugar cane is not native to Africa but is infected by a complex of related virus species (*Sugarcane streak virus, Sugarcane streak Reunion virus, Sugarcane streak Egypt virus *and *Eragrostis streak virus*) which have been isolated from native grasses of the genera *Setaria, Cenchrus, Paspalum *and *Eragrostis*, indicating a wide host range [46]. In contrast, the BEP clade viruses, WDV, BDV and ODV specialize in infecting their respective host plants and thus likely have evolved entirely in the lineage for which they are named [25].

Estimates of short term and longer term evolution rates are available for viruses in the sister genus, *Begomovirus*. Inoculation of plants with infectious cloned DNA of *Tomato yellow leaf curl China virus *(TYLCCV) resulted in the subsequent recovery of viral sequences with substitutions at a frequency of about 10^-4 ^substitutions per site during a 60 day growth period in plants [47]. Consistent with the experimental result, phylogenetic analysis suggested a substitution frequency of 5 × 10^-4 ^per site per year for the related *Tomato yellow leaf curl virus *[17]. Thus on these time scales, the genomes change as frequently as most RNA virus genomes [48,49]. These time scales sample sites that evolve rapidly. The distribution of nucleotide diversity along the WDV genome (Figure 2) shows that areas of the genome with high diversity represent only a small percentage of the total genome. In the TYLCCV study [47], four nucleotide positions accounted for close to half (18 of 41) of the observed substitutions. Thus, it is likely that highly mutable positions gave rise to the substitutions in the *Begomovirus *investigations, while the deeper phylogenetic tree construction employed in our *Mastrevirus *work focuses on the areas of the genome with low nucleotide diversity. These are also the areas subjected to strong purifying selection. The apparent discrepancy between very long-term and short or long-term evolution rates in the *Geminiviridae *is reminiscent of similar findings in the *Tobamovirus *analysis. Understanding this apparent difference awaits further analysis.

## Methods

### Virus isolates

WDV was collected throughout China during field surveys in the growing seasons 2004 to 2006. The 28 isolates described here originated from wheat planted in different agro-ecological areas in China, including the northwestern (Shaanxi and Gansu provinces), northern (Shanxi and Hebei provinces), central (Henan province), and southwestern (Yunan province) areas. All the field isolates were inoculated to the susceptible wheat (*Triticum aestivum *L.) cultivar *Fengkang *No. 8 by vector leafhoppers (*Psammotetix alienus *L.) to increase virus concentration and to allow serological typing or sequencing of polymerase chain reaction (PCR) products. The wheat plants were later tested for WDV with ELISA using an antiserum (Bio-Rad, Marnes la Coquette, France). Leaves were collected from WDV-positive plants displaying typical symptoms of WDV infection, and stored at -80°C. Details of the isolates, their names, provinces of collection, original host plant, and years of collection are shown in additional file [see Additional file 1].

### Cloning of entire genomes

Total DNA was extracted from systemically WDV-infected wheat leaves [50]. DNA extracts were used as template for PCR amplification, performed in a 50 μL reaction solution containing 10×Taq Buffer, 2.5 mM dNTP (each), 0.4 mM of the viral sense and complementary sense primers designed according to the conserved sequences of WDV genomes [see Additional file 3], and Ampli Taq DNA polymerase (Applied Biosystems, Foster City, CA, USA). PCR reactions were carried out for 35 cycles, each consisting of denaturation at 94°C for 1 min, annealing at 55°C for 1 min, and extension at 72°C for 1 min, with 95°C for 2 min at the beginning and 72°C for 10 min at the final step. The expected PCR products were 767 bp, 1152 bp and 1041 bp, using the primer pairs of 40F/806R, 735F/1886R and 1828F/118R, respectively, and together covered the entire length of the viral genome. The PCR product segments were electrophoresed in 1.0% agarose gels, bands were excised using a razor blade and purified using the BioTeq PCR quick Gel Extraction Kit (BioTeq, Inc, USA).

### DNA sequencing

Nucleotide sequences of the entire genome of each isolate were determined using the above PCR fragments. The purified fragments were cloned into the pMD18-T vector (Takara, Dalian, China). The plasmids were transformed into *Escherichia coli *strain JM110 and plasmid DNA was isolated from overnight cultures by alkaline lysis. Insert sequences were determined on at least three clones for each PCR fragment using the dideoxynucleotide chain termination method by an automated sequencer (ABI BigDye 3.1, Applied Biosystems, Foster City, CA). Sequence data were assembled using DNASIS version 3.5 (Hitachi) or BIOEDIT version 5.0.9 [51]. The nucleotide sequence data have been submitted to GenBank databases and assigned accession numbers EF536859 through EF536886.

### Phylogenetic and molecular diversity analysis

Complete genomes of the 28 WDV isolates sequenced in this study and 18 entire sequences of other WDV, BDV and ODV isolates obtained from the NCBI database (National Center for Biotechnology Information, Bethesda, MD, USA) were analyzed. The coding and intergenic regions were annotated by reading frame or following NCBI's annotations. The complete genome sequences of the WDV, BDV and ODV genomes were aligned with CLUSTAL W V.1.8. MEGA V.4.0 [52] determined the number of nucleotide substitutions per site (evolutionary distance) between the strains. Phylogenetic trees were constructed by neighbor-joining (NJ), and maximum parsimony (MP) as implemented by MEGA version 4.0 [52] and DNAPARS of PHYLIP package version 3.5 [53], respectively, based on the Kimura 2-parameter distance matrix model. Bootstrap confidence values were obtained for 1000 replicates (Figure 1). The homologous regions of the genome of an isolate of *Maize streak virus *(MSV) (NC_001346) [54] were used as the outgroup for these analyses, as BLAST searches had shown them to be the sequences in the international sequence databases most closely related to those of MSV. Treemap 4.1.1 [44] was used to test and display the correspondence between plant and virus trees. The Watterson's estimator of θ (**θ**w) [38] and the average pairwise nucleotide diversity Pi(π) [39], were estimated using DnaSP version 4.10.2 [55]. Also, the program was used to estimate the proportions of synonymous and nonsynonymous substitutions by the Jukes-Cantor one-parameter model.

To evaluate the sequence relationships among mastrevirus genomes, a selection of mastrevirus sequences available at the time was obtained [see Additional file 2]. A manually adjusted multiple sequence alignment based on encoded amino acid sequences was generated using Se-Al [56]such that MP-CP and Rep-RepA regions were satisfactorily aligned. These regions were separately excised from the alignment for further manipulation. In cases where multiple sequences were available for the same named virus, a consensus sequence was generated by Se-Al. For examination of host phylogenetic relationships, *rbcL *sequences were retrieved from GenBank/DDBJ/EMBL. They are identified in the legend of Figure 4. These were also aligned. Aligned sequences were examined using PAUP [57] by testing models supplied by Modeltest [58]. The parameters for the best model were used to construct neighbor joining trees as implemented in PAUP. To test for consistency, bootstrapped neighbor joining was also performed using Phylip package programs, Seqboot, DNAdist, Fitch, and Consense [53]. Resulting trees were manually manipulated to minimize tangles between host and virus trees. The validity of a molecular clock for several assemblages of sequences was tested using the log ratio test as described by Posada [59]. To determine selection models acting on the WDV, BDV and ODV genes, nucleotide substitutions at synonymous (K_s_) and non-synonymous (K_a_) positions of genes were calculated by DnaSP version 4.10.2 [55].

### Nucleotide Substitution Frequency and Divergence Times

The average frequency with which mastrevirus sequence sites mutate in evolution is unknown. For highly mutable sites the substitution frequency has been estimated [47] at 3 × 10^-4^/site during a 60 day growing period for *Tomato yellow leaf curl China virus *(TYLCCNV). That value is clearly an overestimate of the average frequency.

Nevertheless, since our interest was in determining the relative ratios of divergence times of WDV, BDV and ODV from MSV, of ODV from WDV and BDV, and of WDV from BDV, the number was used to obtain divergence time estimates with MEGA software [52]. Host divergence times were obtained from literature. Resulting calculated virus divergence times were normalized to 100 for the (WDV-BDV-ODV)-MSV split and values were plotted against corresponding host divergences. Linear regression was used to evaluate the correspondences and to determine an appropriate conversion factor that could be applied, assuming uniformity of the molecular clock, to the relative divergence times of the viruses.

### Detection of recombination and mutation bias

To investigate the extent of recombination within the data set, the aligned sequences were examined using the Recombination Detection Program (RDP3) [41], GENECONV [60], BOOTSCAN [61], MAXIMUM CHISQUARE [62], CHIMERA [41], SISTER SCAN [42] and and phylpro [63] recombination detection methods as implemented in RDP3 [41], (details of program settings available from http://darwin.uvigo.es/rdp/heath2006.zip). The transversion and transition differences of all pairs of sequences were calculated using the discalc program (kindly supplied by G. F. Weiller, Australian National University) and these were compared in diplomo scatter plots [64].

## Authors' contributions

BLW performed molecular methods, gathered sequence information from GenBank and research, conducted bioinformatics analysis, and wrote the manuscript. UM carried out evolution time analysis and the codivergence analysis between virus and host, and edited the manuscript. XFW designed the project, provided tools/reagents and edited the manuscript. XYG designed and conducted bioinformatics analysis. LJF conducted bioinformatics analysis. GHZ aided the study design and provided tools/reagents. All authors read and approved the final manuscript.

## Supplementary Material

Additional file 1 **WDV sequences of wheat isolates from China obtained in this research.** Isolate names, collected times, regions and GenBank accession numbers used in this study. Click here for file

Additional file 2 **Sequences from GenBank for sequence comparision.** The sequences obtained from the NCBI database used in phylogenetic comparison and codivergence analysis. Click here for file

Additional file 3 **The primers used for PCR and corresponding annealing temperature.** Primer's name, sequences and corresponding annealing temperature used in this study. Click here for file
