# Supplementary material for: Assessment of codivergence of Mastreviruses with their plant hosts
Source: BMC Evol Biol. 2008 Dec 18;8:335. doi: 10.1186/1471-2148-8-335 (PMC2630985; doi:10.1186/1471-2148-8-335)
Supplement: Additional file 1 — WDV sequences of wheat isolates from China obtained in this research. Isolate names, collected times, regions and GenBank accession numbers used in this study. [file 1471-2148-8-335-S1.doc]

**Additional file 1**

**Table 1.** WDV sequences of wheat isolates from China obtained in this research.

| **Isolate^a^** | **Collected from** | **Host** | **Accession No.** | **Date** |
| --- | --- | --- | --- | --- |
| WDV-[HBSJZ04] | Shijiazhuang, Hebei | Wheat | EF536862 | 5/2004 |
| WDV-[GSGG05-1] | Gangu, Gansu | Wheat | EF536859 | 4/2005 |
| WDV-[GSGG05-2] | Gangu, Gansu | Wheat | EF536860 | 4/2005 |
| WDV-[SXYC05-2] | Yuncheng, Shanxi | Wheat | EF536875 | 4/2005 |
| WDV-[SXYC05-3] | Yuncheng, Shanxi | Wheat | EF536876 | 4/2005 |
| WDV-[SXYL05-1] | Yangling, Shaanxi | Wheat | EF536877 | 4/2005 |
| WDV-[SXYL05-2] | Yangling, Shaanxi | Wheat | EF536878 | 4/2005 |
| WDV-[SXYL05-3] | Yangling, Shaanxi | Wheat | EF536879 | 4/2005 |
| WDV-[SXYL05-4] | Yangling, Shaanxi | Wheat | EF536880 | 4/2005 |
| WDV-[SXYL05-5] | Yangling, Shaanxi | Wheat | EF536881 | 4/2005 |
| WDV-[SXYL05-6] | Yangling, Shaanxi | Wheat | EF536882 | 4/2005 |
| WDV-[HNZZ05] | Zhengzhou, Henan | Wheat | EF536861 | 5/2005 |
| WDV-[SXTY05-1] | Taiyuan, Shanxi | Wheat | EF536872 | 5/2005 |
| WDV-[SXTY05-2] | Taiyuan, Shanxi | Wheat | EF536873 | 5/2005 |
| WDV-[SXTY05-3] | Taiyuan, Shanxi | Wheat | EF536874 | 5/2005 |
| WDV-[HBSJZ06-3] | Shijiazhuang, Hebei | Wheat | EF536863 | 4/2006 |
| WDV-[HBSJZ06-4] | Shijiazhuang, Hebei | Wheat | EF536864 | 4/2006 |
| WDV-[HBSJZ06-5] | Shijiazhuang, Hebei | Wheat | EF536865 | 4/2006 |
| WDV-[HBSJZ06-6] | Shijiazhuang, Hebei | Wheat | EF536866 | 4/2006 |
| WDV-[HBSJZ06-7] | Shijiazhuang, Hebei | Wheat | EF536867 | 4/2006 |
| WDV-[HBSJZ06-9] | Shijiazhuang, Hebei | Wheat | EF536868 | 4/2006 |
| WDV-[HBSJZ06-10] | Shijiazhuang, Hebei | Wheat | EF536869 | 4/2006 |
| WDV-[HBSJZ06-11] | Shijiazhuang, Hebei | Wheat | EF536870 | 4/2006 |
| WDV-[HBSJZ06-12] | Shijiazhuang, Hebei | Wheat | EF536871 | 4/2006 |
| WDV-[YNKM06-1] | Kunming, Yunnan | Wheat | EF536883 | 4/2006 |
| WDV-[YNKM06-3] | Kunming, Yunnan | Wheat | EF536884 | 4/2006 |
| WDV-[YNKM06-4] | Kunming, Yunnan | Wheat | EF536885 | 4/2006 |
| WDV-[YNKM06-2] | Kunming, Yunnan | Wheat | EF536886 | 4/2006 |

a: All were collected from wheat and fully sequenced.
