# Supplementary material for: Assessment of codivergence of Mastreviruses with their plant hosts
Source: BMC Evol Biol. 2008 Dec 18;8:335. doi: 10.1186/1471-2148-8-335 (PMC2630985; doi:10.1186/1471-2148-8-335)
Supplement: Additional file 2 — Sequences from GenBank for sequence comparision. The sequences obtained from the NCBI database used in phylogenetic comparison and codivergence analysis. [file 1471-2148-8-335-S2.doc]

**Additional file 2**

**Table 2.** Sequences from GenBank for sequence comparison.

| **Access No.** | **Host** | **Collected from** | **Abbreviation** | **Date** |
| --- | --- | --- | --- | --- |
| AM040733 | Wheat | Hungary | WDV-[HU-F] | 28/6/2005 |
| AM040732 | Wheat | Hungary | WDV-[HU-B] | 28/6/2006 |
| NC_003326 | Wheat | Sweden | WDV-[SE1] | 26/9/2006 |
| DQ868525 | Wheat | China,Taiyuan | WDV-[SXTY04] | 20/12/2006 |
| AJ311031 | Wheat | Sweden | WDV-[SE] | 14/11/2006 |
| X82104 | Wheat | France | WDV-[FR] | 18/4/2005 |
| AM296018 | Barley | Germany | BDV-[SxA18] | 2004 |
| AM296019 | Barley | Czech Rep. | BDV-[Cz19] | 2002 |
| AM296020 | Barley | Germany | BDV-[McP20] | 2001 |
| AM296021 | Wheat | Germany | WDV-[BB21] | 2004 |
| AM296022 | Wheat | Germany | WDV-[SxA22] | 2002 |
| AM296023 | Wheat | Germany | WDV-[SxA23] | 2004 |
| AM296024 | Barley | Germany | WDV-[SxA24] | 2002 |
| AM296025 | Oats | Germany | ODV-[SxA25] | 2005 |
| AM411651 | Barley | Germany | BDV-[BaW1] | 2005 |
| AM411652 | Barley | Germany | BDV-[BaW2] | 2005 |
| X02869 | Wheat | Sweden | WDV-[CZ] | 1/7/2005 |
| AJ783960 | Barley | Turkey | BDV-[TR2] | 14/11/2006 |
| NC_001346 | Maize | South Africa | MSV-[SF]^a^ | <1988 |
| AF329881 | Maize | Zimbabwe | MSV-A[MatA]^a^ | 23/3/2004 |
| AF329889 | Maize | South Africa | MSV-[Raw]^a^ | 23/3/2004 |
| AF329888 | Maize | South Africa | MSV-E[Pat]^a^ | 23/3/2004 |
| AF007881 | Maize | South Africa | MSV-[Set]^a^ | 23/3/2004 |
| U20768 | Maize | South Africa | MSV-B[Vaalhart wheat]^a^ | 23/3/2004 |
| NC_001647 | Panicum | South Africa | PanSV-[Karino]^b^ | 10/7/2008 |
| X60168 | Panicum | Kenya | PanSV-[Kenya]^b^ | 18/4/2005 |
| EU224264 | Panicum | Zimbabwe | PanSV-C[ZmGur]^b^, | 29/2/2008 |
| EU224265 | Panicum | Nigeria | PanSV-D[Nifo]^b^ | 29/2/2008 |
| NC_003744 | Sugarcane | South Africa | ScSV^c^ | 10/7/2008 |
| NC_001868 | Sugarcane | Egypt | ScSV-Egypt^c^ | 22/7/2008 |
| NC_004755 | Sugarcane | Reunion | ScSV-Reunion^c^ | 10/7/2008 |
| EU244914 | *Cenchrus myosuroides* | Reunion | ScSV-B[Rpie] ^c^ | 29/2/2008 |
| EU244916 | *Cenchrus myosuroides* | Zimbabwe | ScSV-B[ZmNya] ^c^ | 29/2/2008 |
| EU445692 | *Urochloa plantaginea* | Nigeria | USV-Nlag1^d^ | 23/6/2008 |
| EU445693 | *Urochloa plantaginea* | Nigeria | USV-Niwo ^d^ | 23/6/2008 |
| EU445694 | *Urochloa plantaginea* | Nigeria | USV-Nlag2 ^d^ | 23/6/2008 |
| EU445695 | *Urochloa plantaginea* | Nigeria | USV-Nodo ^d^ | 23/6/2008 |
| EU445696 | *Urochloa plantaginea* | Nigeria | USV-Nile ^d^ | 23/6/2008 |
| EU445697 | *Urochloa plantaginea* | Nigeria | USV-Nipe ^d^ | 23/6/2008 |
| EU445698 | *Urochloa plantaginea* | Nigeria | USV-Neji ^d^ | 23/6/2008 |
| EU445699 | *Urochloa plantaginea* | Nigeria | USV-Neji2 ^d^ | 23/6/2008 |
| NC_001478 | *Digitaria sanguinalis* | Vanuatu | DigSV | 10/7/2008 |
| NC_003493 | Bean | South Africa | BeYDV | 18/7/2008 |
| NC_001466 | *Chloris gayana* | Australia | ChlStrMV | 10/7/2008 |
| AM849096 | Chickpea | Pakistan | ChPCDV | 17/7/2008 |
| EU244915 | *Eragrostis curvula* | Zimbabwe | ESV[ZmGur] | 29/2/2008 |
| NC_003379 | *Miscanthus* | Japan | MisSV | 20/10/2006 |
| NC_003822 | Tobacco | Australia | TYDV | 18/7/2008 |

**^a,b,c,d^These sequence entries contributed, respectively, to consensus sequences for MSV, PanSV, ScSV and USV.**
