# Supplementary material for: Assessment of codivergence of Mastreviruses with their plant hosts
Source: BMC Evol Biol. 2008 Dec 18;8:335. doi: 10.1186/1471-2148-8-335 (PMC2630985; doi:10.1186/1471-2148-8-335)
Supplement: Additional file 3 — The primers used for PCR and corresponding annealing temperature. Primer's name, sequences and corresponding annealing temperature used in this study. [file 1471-2148-8-335-S3.doc]

**Additional file 3**

**Table 3**. The primers used for PCR and corresponding annealing temperature.

| **Primer pairs** | **Sequence from 5**’ **to 3**’ | **Temp. °C** |
| --- | --- | --- |
| 40F | TGAGTGCGCGGAGGCTTTTGG | 55 |
| 806R | TCTGGCATTGCCTGTTTCGG | 55 |
| 735F | TCCGTTCATCGGTCCAGTCCG | 55 |
| 1886R | ACTCCGTAAGCCTCGAATCC | 55 |
| 1828F | TTGCGCTTGATCCGCAGGAG | 55 |
| 118R | AAGCTAAGGCATGGCACACATTTCC | 55 |
